# Health Gains and Financial Protection from Pneumococcal Vaccination and Pneumonia Treatment in Ethiopia: Results from an Extended Cost-Effectiveness Analysis

**DOI:** 10.1371/journal.pone.0142691

**Published:** 2015-12-09

**Authors:** Kjell Arne Johansson, Solomon Tessema Memirie, Clint Pecenka, Dean T. Jamison, Stéphane Verguet

**Affiliations:** 1 Department of Global Public Health and Primary Care, University of Bergen, Bergen, Norway; 2 PATH, Seattle, WA, United States of America; 3 Department of Global Health, University of Washington, Seattle, WA, United States of America; 4 Department of Global Health and Population, Harvard T.H. Chan School of Public Health, Boston, MA, United States of America; London School of Hygiene and Tropical Medicine, UNITED KINGDOM

## Abstract

**Background:**

Pneumonia and pneumococcal disease cause a large disease burden in resource-constrained settings. We pursue an extended cost-effectiveness analysis (ECEA) of two fully publicly financed interventions in Ethiopia: pneumococcal vaccination for newborns and pneumonia treatment for under-five children in Ethiopia.

**Methods:**

We apply ECEA methods and estimate the program impact on: (1) government program costs; (2) pneumonia and pneumococcal deaths averted; (3) household expenses related to pneumonia/pneumococcal disease treatment averted; (4) prevention of household medical impoverishment measured by an imputed money-metric value of financial risk protection; and (5) distributional consequences across the wealth strata of the country population. Available epidemiological and cost data from Ethiopia are applied and the two interventions are assessed separately at various incremental coverage levels.

**Results:**

Scaling-up pneumococcal vaccines at around 40% coverage would cost about $11.5 million and avert about 2090 child deaths annually, while a 10% increase of pneumonia treatment to all children under 5 years of age would cost about $13.9 million and avert 2610 deaths annually. Health benefits of the two interventions publicly financed would be concentrated among the bottom income quintile, where 30–40% of all deaths averted would be expected to occur in the poorest quintile. In sum, the two interventions would eliminate a total of $2.4 million of private household expenditures annually, where the richest quintile benefits from around 30% of the total private expenditures averted. The financial risk protection benefits would be largely concentrated among the bottom income quintile. The results are most sensitive to variations in vaccine price, population size, number of deaths due to pneumonia, efficacy of interventions and out-of-pocket copayment share.

**Conclusions:**

Vaccine and treatment interventions for children, as shown with the illustrative examples of pneumococcal vaccine and pneumonia treatment, can bring large health and financial benefits to households in Ethiopia, most particularly among the poorest socio-economic groups.

## Introduction

Globally, 6.6 million children under 5 years of age were estimated to die in 2012 [1]. Lower respiratory infections are one of the leading causes of death for children under 5, and 10 to 20% [2] of all under-5 deaths have been estimated to be due to pneumonia. Ethiopia is one of the five countries in the world with most child deaths: 205,000 children under 5 were estimated to have died in 2012 [1]. As elsewhere, lower respiratory infections are the main killers. Between 33,000 and 37,000 [3,4] children under 5 die annually from pneumonia in Ethiopia according to recent estimates. Most of these deaths could easily be averted if effective treatment and vaccines were available.

Hard priorities need to be made across diseases and population sub-groups in such low- and middle-income country settings. Little is spent on healthcare in Ethiopia: the total annual health expenditure is of 18 US$ per capita [5], and information on the opportunity cost of health care spending are needed [6]. A large proportion of total health spending in Ethiopia is from out-of-pocket (OOP) expenditures, whose estimates vary between 30 and 40% over the last ten years [5]. At a policy level, priority decisions must balance trade-offs between maximizing population health and equalizing the distribution of health outcomes [7–10], including the prevention of medical impoverishment and the provision of financial risk protection (FRP). A better understanding on how a broad range of such fairness concerns can empirically be incorporated into real world priority decisions is needed [9–11].

Standard cost-effectiveness analysis (CEA) [12] provides important, but incomplete information as a basis for priority setting across health interventions and for designing healthcare packages [6,13]. Information on health inequality among income groups and medical impoverishment are important in addition to cost-effectiveness [8,14]. A methodology of extended cost-effectiveness analysis (ECEA) [15–18] was recently developed to evaluate the consequences of health policy in these domains of equity and FRP, in addition to health improvements, and subsequently applied to the case study of rotavirus vaccination [16]. Rheingans et al. have estimated income-related mortality differentials from scale-up of rotavirus vaccination, and found vaccination of populations with low-income to save most lives and to be most cost-effective [19]. Evaluations of both impact on health and medical impoverishment across income groups are rare. However, such information is crucial in priority setting, especially in Ethiopia where close to 40% of the population lives below the income poverty line (less than 1.25 Purchasing Power Parity (PPP) $ per day as defined by the World Bank) [20] and disease burden is substantial.

Two-thirds of all pneumonia deaths are estimated to be caused by *Streptococcus pneumoniae*, *Haemophilus influenzae* type B (Hib) and the influenza virus [4]. Only about 30% of all children under the age of 5 with symptoms of pneumonia utilize healthcare in Ethiopia [21]. An ambitious national scale-up of a 13-valent pneumococcal conjugate vaccine (PCV-13), a three-dose course vaccine, was initiated nationally in 2011 in Ethiopia with support from the Global Alliance for Vaccines and Immunizations (GAVI) [22]. There is no public information about the vaccine coverage for this program currently available. Vaccine injections are given at months 2, 4 and 12 months, respectively. The cost per dose, US$3.50 (as currently procured to GAVI by manufacturers), has been found to be the main cost-driver in PCV-13 programs, as in Gambia [23]. GAVI now supports US$3.30 per dose, which leads to a substantial price reduction for countries like Ethiopia who co-finance only $0.20 per dose [22]. Concurrently, some health policies in Ethiopia are now also focusing on reducing the burden of pneumonia deaths by scaling up case management of pneumonia [24].

The objective of this paper is to apply ECEA methods to evaluate scale-up and universal public finance (UPF)–government financing of an intervention irrespective of who is receiving it–of pneumococcal vaccine and pneumonia treatment in Ethiopia. With UPF, households would receive either vaccination or treatment for free at the point of care. More specifically, we evaluate the magnitude and distribution of expected health outcomes and FRP provided to individuals in the population by hypothetically increasing coverage of PCV-13 and pneumonia treatment at various coverage levels.

## Methods

**Fig 1:**
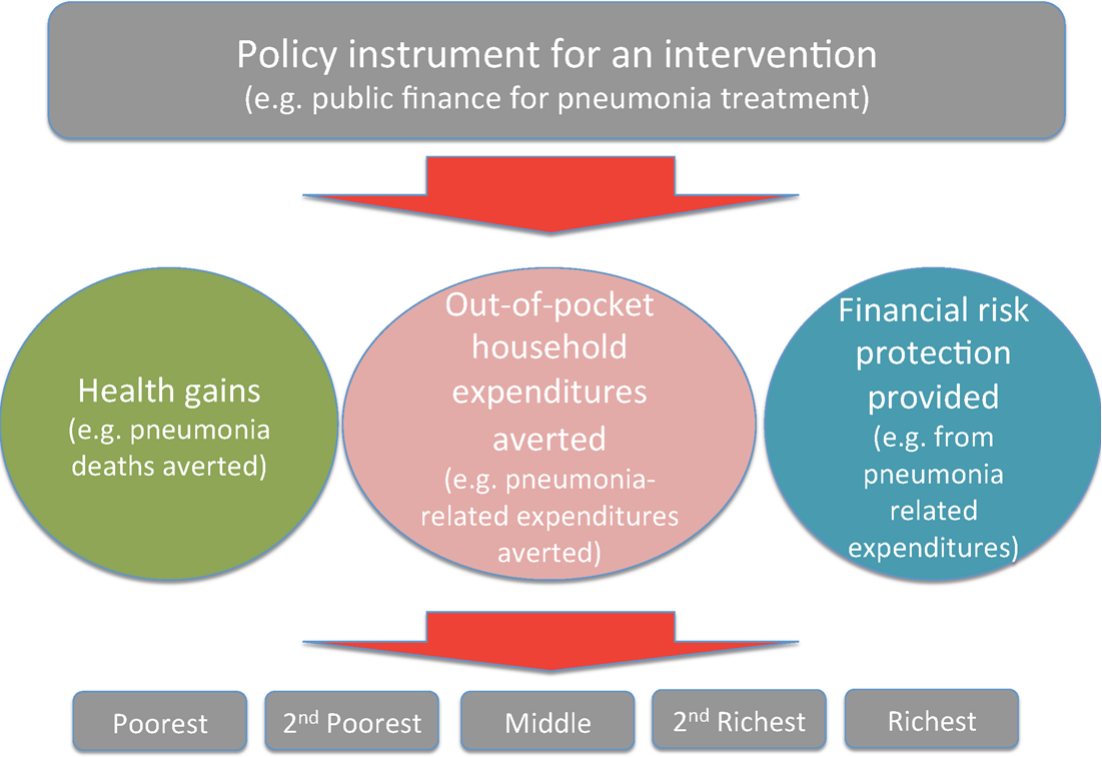
Summary of the conceptual structure of the methodology of extended cost-effectiveness analysis (ECEA) where we measure the program impact in four domains: (1) health gains; (2) household private expenditures averted; (3) prevention of household medical impoverishment; and (4) distributional consequences across the wealth strata of the country population.

We use ECEA methods [15–17] to evaluate the health and non-health impacts of increased coverage due to UPF of PCV-13 and case management of pneumonia. We hypothetically follow the current under-five birth cohort in Ethiopia. Specifically, we estimate the level and distribution (across income groups) of the health benefits by the two publicly financed interventions; the private households’ medical expenditures related to treatment of these diseases averted, and the total costs of the program; and the FRP afforded by the program measured by an imputed money-metric value of insurance provided (see Fig 1 for the conceptual structure of the ECEA methodology). The two interventions are assessed separately: (I1) PCV-13 for newborns; (I2) treatment of pneumonia for under-fives. Values for all parameters are listed and defined in Table 1.

**Table 1:** Parameters used for the economic evaluation of universal public finance (UPF) for pneumococcal vaccination and pneumonia treatment in Ethiopia.

Parameter	Value	Reference
Epidemiology		
Annual number of births	2,800,000	[53]
Population under 5 years	13,840,000	[54]
Deaths due to pneumonia (U5D, annual)	37,300	[4]
Incidence rate of pneumonia <5 years, all causes	0.07	[21]
Episodes of pneumococci pneumonia annually <5years (severe)	240,000 (72,300)	[26]
Pneumonia-U5D attributed to pneumococci	12,300	[26]
Relative risk ratio of mortality, Q 1–5	1.3; 1.1; 0.9; 0.9; 0.8*	[21]
Proportion of women 15–49 currently pregnant, Q 1–5	0.098; 0.085; 0.075; 0.073; 0.045	[21]
Interventions		
Amoxicillin efficacy as case management of pneumonia**	0.7	[35]
Vaccine efficacy (per 3-dose course) against:		
- Pneumonia deaths/episodes (pneumococcal)	0.58	[32]
- Meningitis deaths (pneumococcal)	0.64	[33]
- NPNM deaths (pneumococcal)	0.89	[34]
Coverage of pneumonia treatment (Q 1–5), before UPF	0.16; 0.25; 0.22; 0.33; 0.62	[21]
Coverage of pneumonia treatment (Q 1–5), after UPF	0.26; 0.35; 0.32; 0.43; 0.72	Authors’ assumption
Coverage of pneumococcal vaccine (Q 1–5), before UPF	0; 0; 0; 0; 0	[21]
Coverage of pneumococcal vaccine (Q 1–5), after UPF	0.26; 0.29; 0.31; 0.42; 0.62	Authors’ assumption
Probability of outpatient visit due to pneumonia (Q 1–5)	0.16; 0.25; 0.22; 0.33; 0.62	[21]
Probability of inpatient visit due to pneumonia (Q 1–5)	0.09; 0.09; 0.09; 0.09; 0.09	[37]
Probability of outpatient visit due to meningitis (Q 1–5)	0.75; 0.75; 0.75; 0.75; 0.75	Authors' assumption
Probability of inpatient visit due to meningitis (Q 1–5)	0.75; 0.75; 0.75; 0.75; 0.75	Authors' assumption
Probability of outpatient visit due to NPNM (Q 1–5)	0.75; 0.75; 0.75; 0.75; 0.75	Authors' assumption
Probability of inpatient visit due to NPNM (Q 1–5)	0.75; 0.75; 0.75; 0.75; 0.75	Authors' assumption
Costs		
Hospitalization cost for Pneumonia (2011 US$)	$84	[41]
Hospitalization cost for Meningitis (2011 US$)	$182	[41]
Outpatient clinic visit cost for pneumonia (2011 US$)	$45	[41]
Cost of transportation to a facility (2011 US$)	$7 ***	[38]
Vaccine co-finance by Ethiopian government (per dose, 3 doses needed):		
- No GAVI subsidy	$3.5	[22]
- With GAVI subsidy	$0.2	[22]
Vaccination system cost (2011 US$, per 1 vial course, 3 doses needed)	$0.5	[39]
GDP per capita (2011 US$)	$360	[5]
Gini index	0.3	[5]
Utility function as a function of individual income y	y^(1-r) / (1-r) with r = 3	S1 Text and [15,16,43]

U5D, under-five deaths; NPNM, Non-Peumonia Non-Meningitis; Q = income quintile (1 is poorest; 2 second poorest; 3 middle; 4 second richest; 5 richest)

*Authors’ calculations based on EDHS 2011 [21]

** risk reduction of pneumonia on child deaths

*** the average cost of transportation for last consultation across facilities in 2000 was 14.6 Birr, and this was converted to 2011 US$.

### Health benefits

We use estimates of under-5 deaths (U5D) attributed to pneumonia and pneumococcal disease reported elsewhere [4,25,26]. For all children under 5 years of age in Ethiopia, the following annual assumptions were applied before scale-up of interventions: 37,000 pneumonia-related deaths [4,26] and pneumococcal disease cause 12,000 pneumonia deaths (Table 1) [4,26]. There are 90 different serotypes of pneumococci [27]. However, only a fraction of these serotypes causes invasive disease. Out of all pneumococcal deaths, 90% were due to pneumonia, 7% to meningitis and 3% to other diseases [28].

Following Rheingans et al. [17], we distributed the total under-five deaths due to pneumonia and pneumococcal disease in the country, while using a ‘risk index’ varying by different income groups. Specifically, since pneumonia accounts for a large proportion (around 15%) of under-five deaths [29], we approximately assumed a risk ratio gradient in dying from pneumonia or pneumococcal disease between income groups by the following proxy, by wealth quintile *i*:

$$R_{i}∼\frac{q_{5,i}}{\sum_{k=1}^{5}q_{5,k}/5}$$ (1)

where *q*_5,*i*_ is the under-five mortality in wealth group *i*, as indicated by the Ethiopian Demographic Health Survey (EDHS) 2011 [21]. To our knowledge, there are no studies from sub-Saharan Africa that document the socio-economic gradient of pneumococcal disease burden. The model follows the current birth cohort in Ethiopia and we adjust the ratio *R_i_* by the percentage of women currently pregnant in each wealth quintile as reported in the EDHS 2011 [21]. Depending on disease-specific mortality, intervention coverage and intervention effectiveness, reductions in disease-specific deaths or cases averted are estimated in each income group. The approach is static and therefore not able to capture epidemiological changes like herd immunity and serotype replacement from the vaccine, which could be for example captured in a dynamic transmission model.

As for the pneumonia treatment base case, there is a 10% incremental increase to the current coverage of pneumonia case management across all income groups (Table 1). Average coverage of pneumonia treatment was 27% before UPF [21]. After UPF, coverage is raised to 37% (on average). Health gains were calculated for the 10% incremental increase. The PCV-13 base case is scaled up from 0% coverage to the Diphteria-Pertussis-Tetanus, 3^rd^ dose (DPT3) coverage level of the country (38% on average) [21]. DPT3 coverage is meant to reflect a plausible health system capacity and could be used to estimate the fraction of newborns that would receive all three doses of PCV-13 in the longer term. Less ambitious and more ambitious coverage targets for the two policies are assessed as competing policy options.

PCV-13 protects against the 13 serotypes (1, 3, 4, 5, 6A, 6B, 7F, 9V, 14, 18C, 19A, 19F, 23F) that are typically associated with invasive diseases like pneumonia, sepsis and meningitis. These 13 serotypes have been estimated to cause around 70% of all pneumococcal invasive disease in GAVI-eligible countries [30]. One meta-analysis of PCV-9 and PCV-11 in African settings found a 26% reduction in all-cause pneumonia-related deaths of children less than five years of age [31]. The affected fraction of pneumococcal deaths are 33% of all pneumonia deaths [4]. Drawing on the literature, we assumed a 58% cause-specific mortality reduction and episode reduction of the vaccine for pneumococcal pneumoniae [32], 64% for pneumococcal meningitis deaths [33], and 89% for non-pneumonia-non-meningitis (NPNM) pneumococcal deaths [34]. Serotype distribution is implied in the vaccine effectiveness. This assumption is supported by comparing the estimate of PCV-13 efficacy with the cumulative proportion of invasive pneumococcal disorders caused by the PCV-13 serotypes. PCV-13 serotypes account for around 70% of invasive pneumococcal disorders [30] in most African regions and PCV-13 efficacy is around 60% for pneumococcal related deaths [32].

The effectiveness of community-based case management of pneumonia draws on a meta-analysis of studies from Africa, and amoxicillin was found to reduce pneumonia-related deaths by 70% [35]. In this analysis, antibiotic acts on preventing all kinds of pneumonia deaths, the pneumococci-related deaths as well those related to other pathogens (e.g. Hib). We used 70% as effectiveness against all kinds of pneumonia deaths [35]. By case management we mean that community health workers follow standard clinical algorithms/guidelines [36] to classify respiratory illness and start with oral amoxicillin for mild/moderate cases of pneumonia (fast breathing and/or chest indrawing) and refer severe cases to health facilities for injectable antibiotics and other supportive care.

### Consequences for household private expenditures and health systems

Pneumonia and pneumococcal disease, whether fatal or non-fatal, impose an economic and financial burden on health systems and families and households. Before the introduction of the program, we assume that individuals privately pay at a 34% level for treatment of pneumonia (the proportional OOP expenditures on health services in Ethiopia according to estimates by the World Health Organization (WHO)) [5]. The remaining 66% is covered by the government, the demand of which varies by income group. The current coverage of basic vaccines in Ethiopia is low (S1 Table) [21]. Substantial investments into the routine immunization program are needed in order to succeed with high coverage of pneumococcal vaccines. A crude estimate of introduction costs for strengthening the existing routine immunization program was therefore done (see S1 Text for details on calculations). Annual government cost of maintaining current coverage and incremental cost to reach a 90% coverage level for all vaccines were estimated separately, and then summed up to yield total introduction cost (S1 Table).

We estimate the amount of household private expenditures averted for each income quintile by UPF of pneumonia treatment and PCV-13. We quantify what households would pay due to illness-related cost in the absence of the program. Pneumonia-related private expenditures depend on the incidence and probability of seeking care of severe and less severe cases of all-cause pneumonia and pneumococcal pneumoniae (Table 1) [4,21,26,37]. Severe cases are treated as inpatients and less severe cases are treated as outpatients in the model. As we only had data on access to outpatient care of pneumonia, we had to make assumptions for the other types of care. Both interventions represent cost savings from the household perspective. After UPF of pneumonia treatment takes place, individuals would pay 0% of the treatment costs, the government would pay 100% of the treatment costs. After UPF of the vaccine is introduced, individuals pay 0% of vaccine costs. Vaccine efficacy and their reduction of incidence rates have an indirect impact on the size of private expenditures. Vaccines reduce private household expenditures since vaccines lower the risk that a child gets a pneumococcal disorder (and the OOP expenditures associated with inpatient and outpatient care). In addition, pneumococcal vaccination indirectly reduces the household risk of experiencing transport costs since pneumococcal disorders are prevented. The baseline cost of transportation to a facility was on average 14.6 Ethiopian Birr in 2000 [38], which converts to $6.8 in 2011.

We estimate the total incremental cost to the government of increased coverage of each program. Vaccine costs are estimated by multiplying the incremental coverage (38% of births) with average system costs per injection (0.5 US$) [39] and size of co-financing per dose (3.5 US$ per dose with no GAVI subsidies (base case) and 0.2 US$ per dose with full GAVI subsidies) [22]. Average system costs per vaccinated child include cold storage, transport, training and public communication. The GAVI subsidies are actually greater than $3.30 per vial because of the additional collective US$1.5 billion payments made under the Advanced Market Commitment [40]. Governmental savings of the vaccine program are included by taking into account effects on reduced treatment demand for pneumococcal disorders (pneumonia, meningitis, non-pneumonia and non-meningitis). Treatment costs for pneumonia treatment are estimated by adding: (1) the costs of reducing current OOP expenditures due to pneumonia treatment from 34% to 0%; and (2) the costs of an incremental scale-up of UPF of pneumonia treatment by 10 percentage points. Average cost per hospitalized case and outpatient case are from Stack et al. [41] (Table 1), and lowest estimates are used. Before UPF, the health system finances 66% of unit costs and patients 34% of unit costs. Estimates by Stack et al. [41] are lower than what was found in a primary study from Zambia [42], where the cost per out-patient visit for pneumonia was $48 and the cost per bed day was $215 (2006 US$).

### Financial risk protection and money-metric value of insurance provided

We use an approach described in great detail elsewhere [15,16] and in the supplementary data (S1 Text). Specifically, we apply a standard utility-based model where risk-averse individuals value protection from the risk of uncertain rare events. UPF provides FRP benefits to households or ‘insures’ households by averting the otherwise expected OOP expenditures associated with pneumonia and pneumococcal disease. FRP benefits of health policies can be measured in different ways. Direct estimates of the households’ private OOP expenditures averted by the policy is one FRP metric; another is to estimate the number of cases of poverty averted (i.e. counting the number of individuals no longer falling under a poverty line because of large OOP expenditures). Here, we use a money-metric value of insurance provided by UPF as our FRP metric [15,43], which attempts to quantify ‘insurance risk premiums’. Practically, we first estimate the individual’s expected income before UPF, depending on disease incidence, treatment utilization and associated costs; then we determine the individual’s ‘certainty equivalent’ by assigning individuals a utility function that specifies their risk aversion, where we estimate the amount of money the individual is willing to have in order to obtain certainty in the outcome (e.g. potential OOP expenditures due to disease). Finally, we derive a money-metric value of insurance (or risk premium) provided by the program by calculating the difference between the expected value of income and the certainty equivalent [15,43]. The FRP metric is subsequently aggregated at the societal level by using an income distribution in the population (with a proxy based on country gross domestic product per capita and Gini coefficient [44]).

We take a health system perspective when estimating the governmental cost of both policies on the one hand and separately add a household perspective when assessing FRP and private household expenditures averted on the other hand. For the vaccine intervention, the time horizon is through the five years of one birth cohort as benefits of vaccination largely occur within the first five years of life, and an annual cross-sectional time horizon is applied for the total population under five years of age that receive pneumonia treatment. No discounting is used. The currency year is 2011 and the consumer price index by each year for Ethiopia is used for inflation-adjustments [5]. The uncertainty in all the parameters in this analysis is high. We therefore pursued a one-way sensitivity analysis with 20–30% increase or reduction of base case values for all the parameters.

S1 Text describes the methods in great detail. All analyses were conducted using the R statistical software (www.r-project-org).

## Results

Total government cost for the pneumonia treatment base case is $13.9 million annually; this includes full public finance for those that currently have access to treatment and a 10% increase in coverage (Table 2). Total vaccine cost for the base case is $11.5 annually; this includes full public finance of vaccines at a DPT-3 level and vaccine prices at $3.5 per dose (Table 2). The annual governmental and health systems savings from vaccines is $820,000, and is mainly due to the expected decrease in pneumococcal disease treatment demand from vaccine effects. A strengthened routine immunization program would be crucial for a successful scale-up of PCV. Introduction cost for strengthening the current routine immunization program in Ethiopia is around $23.3 million annually (S1 Table). Maintenance of current coverage and increased coverage at 90% for all basic vaccines are included in the introduction cost. Summed up, the expected total cost is around $50 million annually for a comprehensive child health program in Ethiopia that includes 90% coverage of routine immunization, and base case of PCV-13 and pneumonia treatment.

**Table 2:** Total government intervention costs, household expenditures averted, deaths averted, and financial risk protection, for each of the two policies (pneumonia treatment and pneumococcal vaccines) provided by universal public finance at different coverage levels in Ethiopia.

	Government intervention cost (2011US$)	Household expenditures averted (2011US$)	Deaths averted	Financial risk protection (2011US$ value of insurance)
**Pneumococcal conjugate vaccine**				
DPT3 coverage	11 503 000	578 000	2 960	30 300
10% coverage across income groups	3 152 000	158 000	810	8 300
80% coverage across income groups	25 212 000	1 266 000	6 480	66 300
90% coverage across income groups	28 363 000	1 424 000	7 290	74 600
**Antibiotic treatment for pneumonia**				
Maintaining current coverage	12 364 000 (no UPF)18 677 000 (UPF)	1 831 000*	0*	197 000*
10% incremental coverage	13 937 000	1 831 000	2 610	197 000
80% coverage across all income groups	67 306 000	1 831 000	20 890	197 000
90% coverage across all income groups	74 930 000	1 831 000	23 500	197 000

UPF = Universal Public Finance

* By switching from non-UPF to UPF

**Fig 2:**
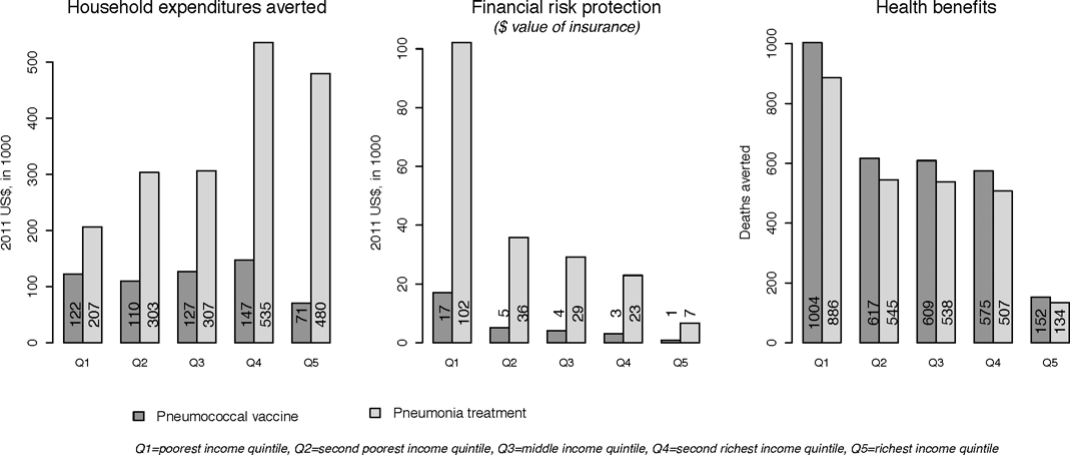
Level and distribution of household expenditures averted, health benefits (deaths averted and severe episodes of pneumococcal pneumoniae averted), and financial risk protection, for scale-up of pneumococcal vaccination and pneumonia treatment provided by universal public finance in Ethiopia.

Pneumococcal vaccines at a DPT-3 level would avert 2,960 deaths annually among all children less than five years of age (Table 2). The pneumonia treatment base case would avert 2610 under-five deaths. In addition, pneumococcal vaccines are estimated to prevent around 60,000 episodes of pneumococcal related pneumonia annually, where 18,000 of these are severe episodes. Both programs save more lives among the poorest groups due to higher disease burden in this population; where between 30 and 40% of all lives saved occur in the poorest quintile (Fig 2). The pro-poor health benefits stands in contrast to the distribution of household private expenditures averted (Fig 2) (linked to the heterogeneous nature of treatment seeking behavior across income groups). We assumed UPF for the program, and this would lead to a decrease in the financial burden for families. Specifically, reductions in household private expenditures occur for those that currently have access to care and there is no impact on private expenditures for those with no access. As expected, wealthier people avert more private expenditures (around 60% of total private expenditures averted from UPF of pneumonia treatment would be felt in the two richest quintiles).

Fig 2 shows the progressive distribution of aggregated FRP afforded, measured as a money-metric value of insurance. Both base case programs offer between 50% and 60% of the total FRP to the poorest quintile. There is a shift in gradients between private expenditures averted and FRP, where the poorest have in absolute terms the lowest private expenditures averted but benefit from the highest FRP. The high FRP among the poorest groups is due to the large expected decline in private expenditures relative to income.

**Fig 3:**
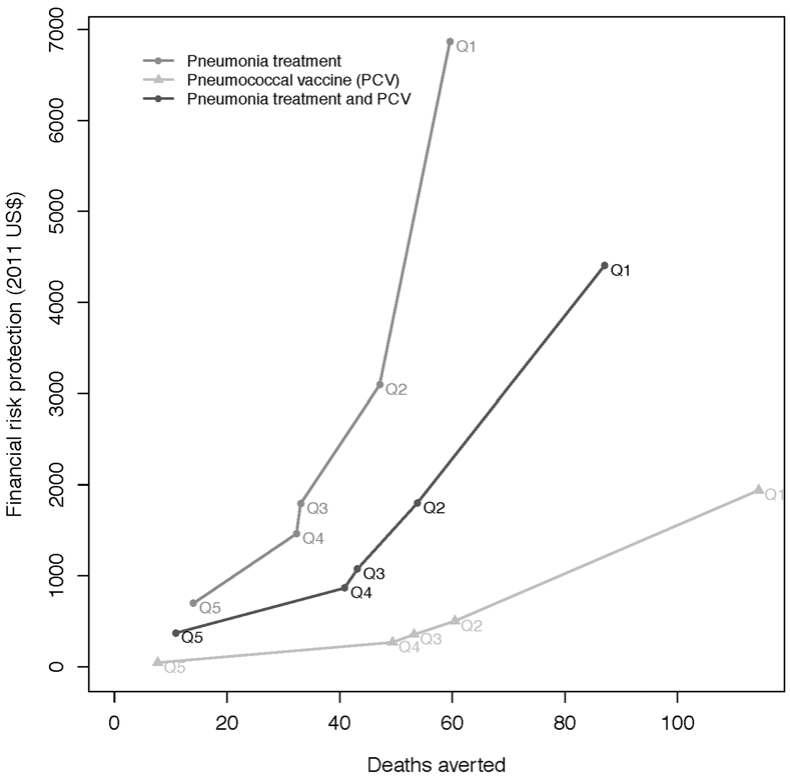
Expected health benefits (deaths averted) versus financial risk protection afforded (2011 US$), per $1,000,000 spent for universal public finance of pneumococcal vaccination and/or pneumonia treatment scale-up in Ethiopia, where results are shown for 5 income quintiles (I is poorest and V is richest).

Vaccines and treatment offer an efficient purchase of health benefits, household expenditures averted and financial risk protection. Examining results per dollar expenditure, a budget constraint of $1 million is introduced (Fig 3). The two dimensions of expected health gains (deaths averted) and FRP afforded are given for the five income groups. The pneumococcal vaccine program saves 280 lives and the pneumonia treatment program saves 190 lives per $1 million spent (Fig 3). A large proportion of the budget for pneumonia treatment is targeted to reduce OOP expenditures for those that are already receiving pneumonia treatment. Both programs are expected to give FRP of $17,000 per $1 million spent.

UPF of vaccines costs around $40 per year of healthy life and UPF of pneumonia treatment costs $107 per year of healthy life in our analysis when we apply the assumption that the life of one child equals 50 years of healthy life.

As expected, the impacts of the two interventions vary according to target coverage (Table 2). An ambitious policy is estimated to cost around $125 million, prevent around $3 million in private expenditures, avert around 30,000 deaths and to give FRP of close to $300,000 annually, where UPF of all interventions are scaled up to a 90% coverage level. As we see from Table 2, increased coverage of pneumonia treatment does not prevent or add any incremental private expenditures or FRP. This is because the scaled-up treatment is offered to patients that do not utilize services in the first place, and therefore there will be no private expenditures averted. Variations in other parameters also impact results.

**Fig 4:**
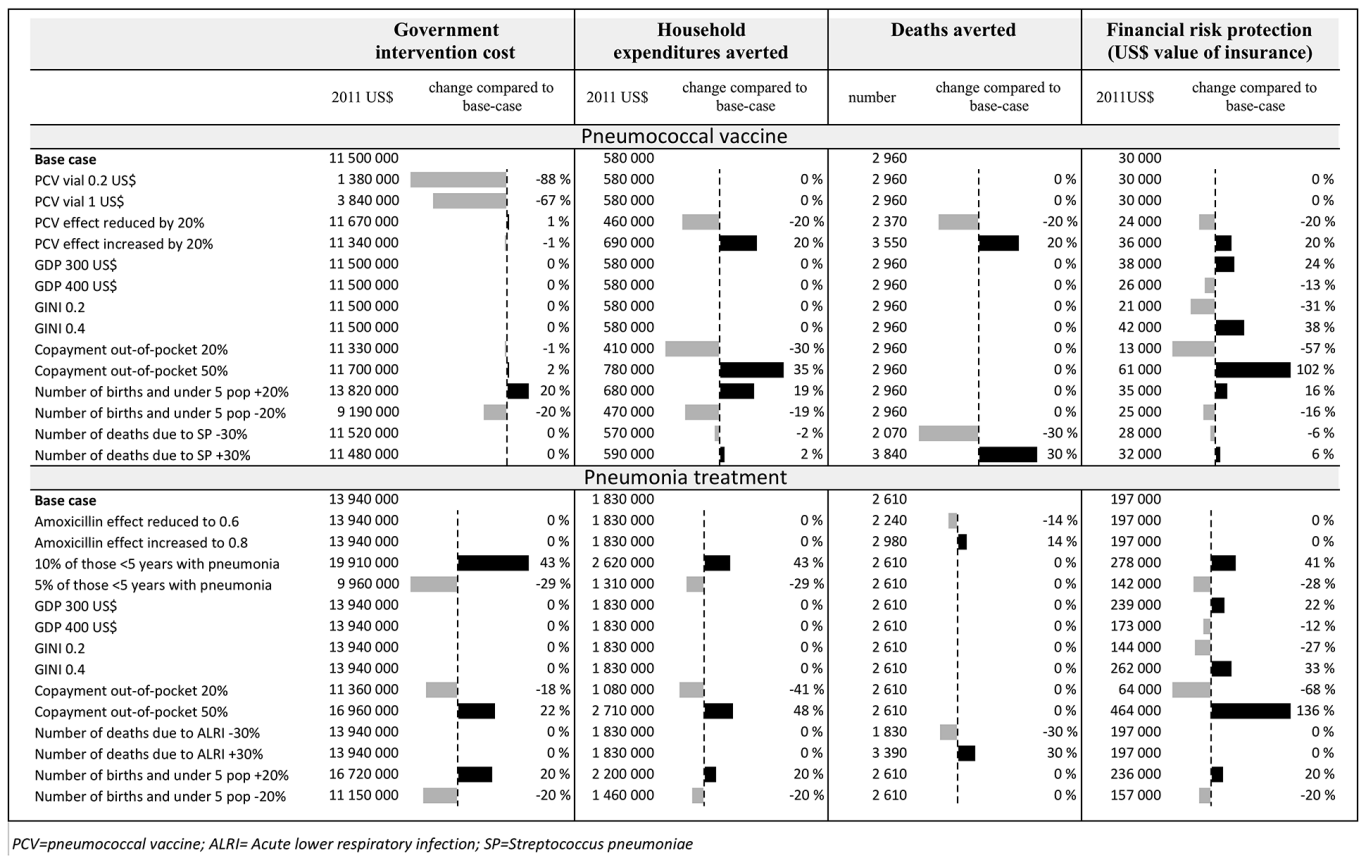
Uncertainty analysis for each of the two policies (universal public finance of pneumonia treatment and pneumococcal vaccines) in Ethiopia, key variables are modified as a one-way deterministic sensitivity analyses, (for more detailed results across income quintiles see S2–S4 Tables).

Total vaccine program costs vary according to the size of GAVI subsidies applied in the analysis (Fig 4). If $1 or $0.2 per dose is applied, total annual cost of the vaccine program is reduced to $3.8 million and $1.4 million, respectively. The co-financing strategy for vaccine prices offered by the GAVI Alliance makes vaccines substantially more cost-effective at a country level. Per $1 million spent, a co-finance of $0.2 per vaccine dose gives a total 2,500 deaths averted and FRP of $27,000, and a co-finance of $1 per dose gives a total 870 deaths averted and FRP of $9,400. Notably, variations in the incidence of pneumonia largely influence the costs of treatment: a 3% increment in incidence of pneumonia increases the annual government cost of the pneumonia treatment program by $6 million (Fig 4). The size of the out-of-pocket copayment has a considerable effect on the expected FRP for both interventions: copayment of 20% reduces FRP by 57% for pneumococcal vaccines and 68% for pneumonia treatment and copayment of 50% gives around a twofold increase in FRP for both interventions (see Fig 4 and S2–S4 Tables for a full set of results of the sensitivity analysis).

## Discussion

This study demonstrates the application of ECEA [15] that enables to measure the size of both health gains and FRP provided for different wealth groups from scale-up of pneumococcal vaccination and pneumonia treatment in Ethiopia. The program gives substantially higher health benefits and FRP for the poor. These measures are useful starting points for an evidence-based discussion on health priorities across policies and interventions [17].

The estimated cost-effectiveness of PCV-13 in our study is comparable to findings in other CEAs. A standard CEA from Kenya found that PCV-10 and PCV-13 is highly cost-effective, at a cost of 59 US$ per Disability Adjusted Life Year averted (around 2000 US$ per live saved) [45]. Program cost is an important factor for the cost-effectiveness of PCV-13. Introduction of PCV-13 into the existing routine immunization program in Ethiopia would require a strengthening of the existing program. The incremental cost of maintaining current coverage and to increase coverage to 90% for all basic vaccines was estimated to be around $23.3 million annually. Since we did not evaluate the expected health benefits from a strengthened routine immunization program, it is not possible to estimate how such increase in introduction costs exactly influence the total cost-effectiveness ratio of PCV-13. This requires further analysis.

However, the ECEA methodology used here goes beyond conventional CEA. Three additional elements are key outcomes in this ECEA: (1) direct impact on household private expenditures; (2) financial risk protection; and (3) the distributional impact of health benefits, (1) and (2). All these dimensions are important equity concerns [46] that we are able to separate out quantitatively. We found that UPF of pneumococcal vaccines and pneumonia treatment are pro-poor policies, as there are higher FRP and more deaths averted in the lowest income groups. Therefore, UPF of pneumonia treatment and pneumococcal vaccines can be efficient policies in a number of dimensions. Financial risks and health risks are transferred to and pooled by governments or donors with UPF. The progressive distribution of health benefits and FRP is relevant for policymakers when setting priorities between pneumococcal vaccines, pneumonia treatment and other interventions for an essential health care package in Ethiopia. If efficient purchase of financial protection and health benefits for the poor is of importance, the findings of this study support that UPF of vaccines and pneumonia treatment should be of high priority in the essential health care package in Ethiopia. The results show that both interventions are progressive prioritizations as interventions are most cost-effective in the poorest groups, which is also confirmed by the high FRP among poor patients per $ invested.

Interestingly, the higher income groups have more private expenditures averted from these policies since access to healthcare is higher in these groups and UPF will minimize those OOP expenditures. However, there is less disposable income among the poorest, and this is the main reason why we observe a shift in the gradient between poor and rich groups when we switch from private expenditures averted to FRP provided by the program. The lower income groups have higher health benefits even if the coverage is highest in the richest income groups. The progressive distribution of health benefits is due to the fact that disease burden is much higher in low-income groups. We did not test targeting policies of the poor because of the political and ethical controversies surrounding such policies [47] and the fact that there is limited evidence documenting that targeting actually benefits the poor.

Lower respiratory infections are complex. Children often get infected several times a year, only a few cases end up as severe infections and multiple pathogens are involved. This complexity should be reflected in disease models. We draw on results from other primary studies to capture this complexity. However, the observational studies or clinical trials we rely on have several limitations. One major limitation is that pathogens are rarely identified in the studies and the distributions of pathogens in Ethiopia are not well known. Case fatality rate and the effectiveness of PCV-13 and amoxicillin vary between pathogens, and this may alter our findings. Case fatality may also vary across income groups. We assume that pneumococcal deaths follow the same income gradient as all <5 deaths [19] because we could not identify any epidemiological studies from Africa that report pneumococcal deaths across income groups. Even if pneumonia and pneumococcal deaths account for a large proportion of all <5 deaths [26], this is a limitation of the model. It is important to address the income gradient of pneumococcal disease burden, as well as other diseases, in future epidemiological studies.

The sensitivity analysis illustrates the substantial impact GAVI subsidies have on health benefits and FRP (Fig 4). It seems plausible, from an Ethiopian perspective, to apply the subsidized vaccine prices in a policy decision on whether to scale up vaccines or not. Global development aid to such subsidies therefore has a tremendous impact on national estimates, and we see that this yields substantially higher benefits for the poorest groups.

There are limitations of our model. First, we do not have disease-specific data on OOP expenditures in Ethiopia due to pneumonia and pneumococcal disease. Second, incidence distribution of various pneumococcal serotypes in Ethiopia is not taken into account and we assume that PCV-13 targets the most lethal and common serotypes in Ethiopia. There is some evidence showing that PCV-13 protects against 74%–88% of all invasive pneumococcal serotypes globally [30]. It has been shown limited variability of protection of PCV-13 across regions [30], which support the accuracy of PCV-13 for an Ethiopian setting. Third, since this is a static model, the model cannot take into account the indirect effects on mortality of serotype replacement in the scale-up of PCV-13. However, some evidence question the impact on mortality of serotype replacement due to lower invasiveness seen by replacing serotypes [48]. Fourth, herd immunity of PCV-13 was not taken into account. Studies conducted in high-income settings showed positive indirect benefits to unvaccinated children, but the attributable fraction of herd immunity on the total mortality reduction from PCV-13 is uncertain and is likely to vary by setting [31,49]. The inclusion of herd immunity into the model would increase both the health and financial benefits of the pneumococcal vaccines. Indeed, in spite of requiring larger amounts of data, there is an important need for economic evaluations using more complex models such as dynamic compartmental of infectious diseases [50,51] which can capture patterns of social mixing, inter-generational effects and herd immunity more generally. Fifth, the impact of PCV on FRP and deaths averted may be underestimated as we only include total benefits from those that receive all three PCV injections. The potential benefits from the 1st and 2nd dose are not included as there is limited data on these effects.

## Conclusion

The current economic growth in Ethiopia (estimated at 7.5% in 2013 [52]) opens for increasing domestic investments in public finance of pneumococcal conjugate vaccination and pneumonia treatment for children. Our results indicate that such investments entail a low risk where there is a high probability that many children will be saved and that these policies can prevent many families from facing impoverishing health expenditures. UPF of vaccines and treatment should therefore be made universally available before less cost-effective interventions are introduced into the Ethiopian health system. The ECEA approach captures financial risk protection and equity into the economic evaluation of vaccine policy, and enables the selection of vaccine packages based on the efficient purchase of both health and financial risk protection benefits. With ECEA, investments in vaccine policy could further be analyzed comparatively with policy investments in other development sectors. Such cross-sectorial analyses are highly relevant for the Ethiopian Ministry of Development.

## Supporting Information

S1 Table Key parameters used to assess the introduction costs (2011 US$) needed to strengthen the basic child immunization program in Ethiopia. (DOCX)

S2 Table Uncertainty analysis of the impact on household expenditures (2011 US$) averted across income quintiles for each of the two policies in Ethiopia (pneumonia treatment and pneumococcal vaccines), key variables are modified as a one-way sensitivity analyses (Q1 is poorest and Q5 is richest; colors identify variances of values, where black cells are the 10% highest values and grey cells are the 10% lowest values). (DOCX)

S3 Table Uncertainty analysis of the impact on deaths averted across income quintiles for each of the two policies in Ethiopia (pneumonia treatment and pneumococcal vaccines), key variables are modified as a one-way sensitivity analyses (Q1 is poorest and Q5 is richest; colors identify variances of values, where black cells are the 10% highest values and grey cells are the 10% lowest values). (DOCX)

S4 Table Uncertainty analysis of the impact on financial risk protection (2011 US$) across income quintiles for each of the two policies in Ethiopia (pneumonia treatment and pneumococcal vaccines), key variables are modified as a one-way sensitivity analyses (Q1 is poorest and Q5 is richest; colors identify variances of values, where black cells are the 10% highest values and grey cells are the 10% lowest values). (DOCX)

S1 Text Details of the Extended Cost Effectiveness Methods used for estimating deaths averted, private expenditures averted, financial risk protection for public finance for pneumococcal vaccination and pneumonia treatment in Ethiopia. (DOCX)
