# Supplementary material for: Health Gains and Financial Protection from Pneumococcal Vaccination and Pneumonia Treatment in Ethiopia: Results from an Extended Cost-Effectiveness Analysis
Source: PLoS One. 2015 Dec 9;10(12):e0142691. doi: 10.1371/journal.pone.0142691 (PMC4674114; doi:10.1371/journal.pone.0142691)
Supplement: S1 Table — (DOCX) [file pone.0142691.s001.docx]

**S1 Table:** Annual government cost of the basic vaccine program in Ethiopia for maintaining current coverage and incremental cost to reach a 90% coverage level for all vaccines.

| **Vaccine** | **Current coverage *** | **Vaccine and injection supply cost per dose (2011US$) **** | **Cost per vaccine vial (2011US$) ***** | **Number of doses/vials per course** | **Government cost of maintaining current coverage (2011US$, in 1,000)** | **Incremental government cost, 90% coverage (2011US$, in 1,000)** |
| --- | --- | --- | --- | --- | --- | --- |
| BCG (at birth) | 66 % | 0.20 | 0.12 | 1 | 590 | 350 |
| DPT-HepB-HiB (at 4 weeks) | 64 % | 0.52 | 1.85 | 1 | 4 210 | 1 760 |
| DPT-HepB-HiB (at 8 weeks) | 52 % | 0.52 | 1.85 | 1 | 3 480 | 2 490 |
| DPT-HepB-HiB (at 12 weeks) | 37 % | 0.52 | 1.85 | 1 | 2 420 | 3 550 |
| Polio (0-2 weeks) | 20 % | 0.05 | 0.13 | 1 | 100 | 460 |
| Polio (4 weeks) | 82 % | 0.05 | 0.13 | 1 | 410 | 40 |
| Polio (8 weeks) | 70 % | 0.05 | 0.13 | 1 | 350 | 100 |
| Polio (12 weeks) | 44 % | 0.05 | 0.13 | 1 | 220 | 230 |
| Measles vaccine | 56 % | 0.47 | 0.35 | 1 | 1 280 | 1 240 |

BCG=Baccillus Calmette-Guérin; DPT-HepB-HiB=Diphtheria-tetanus-pertussis-HepatitisB-Haemophilus influenzae type b; UPF=Universal Public Finance

* Source: Central Statistical Agency [Ethiopia], ICF International (2011) Ethiopia Demographic and Health Survey. Addis Ababa, Ethiopia and Calverton, Maryland, USA: Central Statistical Agency and ICF International.

** Source: Griffiths UK, Korczak VS, Ayalew D, Yigzaw A (2009) Incremental system costs of introducing combined DTwP-hepatitis B-Hib vaccine into national immunization services in Ethiopia. Vaccine 27: 1426-1432.

Average system costs per vaccinated child include cold storage, transport, training and public communication.

*** Source: PAHO, Expanded program of immunization vaccine prices for year 2014
