# Supplementary material for: Health Gains and Financial Protection from Pneumococcal Vaccination and Pneumonia Treatment in Ethiopia: Results from an Extended Cost-Effectiveness Analysis
Source: PLoS One. 2015 Dec 9;10(12):e0142691. doi: 10.1371/journal.pone.0142691 (PMC4674114; doi:10.1371/journal.pone.0142691)
Supplement: S2 Table — (DOCX) [file pone.0142691.s002.docx]

**S2 Table:** Uncertainty analysis of the impact on household expenditures (2011 US$) averted across income quintiles for each of the two policies in Ethiopia (pneumonia treatment and pneumococcal vaccines), key variables are modified as a one-way sensitivity analysis (Q1 is poorest and Q5 is richest; colours identify value variance, where black cells are the 10% highest values and grey cells are the 10% lowest values).

|  | **Pneumococcal vaccine** | | | | | |  | **Pneumonia treatment** | | | | | |
| --- | --- | --- | --- | --- | --- | --- | --- | --- | --- | --- | --- | --- | --- |
|  | **Q1** | **Q2** | **Q3** | **Q4** | **Q5** | **Total** |  | **Q1** | **Q2** | **Q3** | **Q4** | **Q5** | **Total** |
| Pneumonia treatment at 0% coverage |  |  |  |  |  |  |  | 206 669 | 303 272 | 306 670 | 534 906 | 479 612 | 1 831 129 |
| PCV 10% incremental coverage | **33 547** | **30 169** | **34 820** | **40 311** | **19 376** | **158 223** |  |  |  |  |  |  |  |
| **Pneumonia treatment 10% incremental coverage** | |  |  |  |  |  |  | 206 669 | 303 272 | 306 670 | 534 906 | 479 612 | 1 831 129 |
| **PCV coverage at DPT3 level** | 122 448 | 110 118 | 127 094 | 147 135 | 70 721 | 577 516 |  |  |  |  |  |  |  |
| PCV 80% incremental coverage | 268 379 | 241 354 | 278 563 | 322 487 | 155 005 | 1 265 788 |  |  |  |  |  |  |  |
| PCV 90% incremental coverage | 301 926 | 271 523 | 313 383 | 362 798 | 174 381 | 1 424 011 |  |  |  |  |  |  |  |
| Pneumonia treatment 80% incremental coverage | |  |  |  |  |  |  | 206 669 | 303 272 | 306 670 | 534 906 | 479 612 | 1 831 129 |
| Pneumonia treatment 90% incremental coverage | |  |  |  |  |  |  | 206 669 | 303 272 | 306 670 | 534 906 | 479 612 | 1 831 129 |
| PCV vial 0.2 US$ | 122 448 | 110 118 | 127 094 | 147 135 | 70 721 | 577 516 |  | 206 669 | 303 272 | 306 670 | 534 906 | 479 612 | 1 831 129 |
| PCV vial 1 US$ | 122 448 | 110 118 | 127 094 | 147 135 | 70 721 | 577 516 |  | 206 669 | 303 272 | 306 670 | 534 906 | 479 612 | 1 831 129 |
| Amoxicillin effect reduced to 0.6 | 122 448 | 110 118 | 127 094 | 147 135 | 70 721 | 577 516 |  | 206 669 | 303 272 | 306 670 | 534 906 | 479 612 | 1 831 129 |
| Amoxicillin effect increased to 0.8 | 122 448 | 110 118 | 127 094 | 147 135 | 70 721 | 577 516 |  | 206 669 | 303 272 | 306 670 | 534 906 | 479 612 | 1 831 129 |
| PCV effect reduced by 20% | 97 958 | 88 094 | 101 675 | 117 708 | 56 577 | 462 013 |  | 206 669 | 303 272 | 306 670 | 534 906 | 479 612 | 1 831 129 |
| PCV effect increased by 20% | 146 937 | 132 141 | 152 513 | 176 562 | 84 865 | 693 019 |  | 206 669 | 303 272 | 306 670 | 534 906 | 479 612 | 1 831 129 |
| 10% of those <5 years with pneumonia | 122 448 | 110 118 | 127 094 | 147 135 | 70 721 | 577 516 |  | 295 241 | 433 245 | 438 100 | 764 151 | 685 160 | 2 615 898 |
| 5% of those <5 years with pneumonia | 122 448 | 110 118 | 127 094 | 147 135 | 70 721 | 577 516 |  | 147 621 | 216 623 | 219 050 | 382 075 | 342 580 | 1 307 949 |
| GDP 300 US$ | 122 448 | 110 118 | 127 094 | 147 135 | 70 721 | 577 516 |  | 206 669 | 303 272 | 306 670 | 534 906 | 479 612 | 1 831 129 |
| GDP 400 US$ | 122 448 | 110 118 | 127 094 | 147 135 | 70 721 | 577 516 |  | 206 669 | 303 272 | 306 670 | 534 906 | 479 612 | 1 831 129 |
| GINI 0.2 | 122 448 | 110 118 | 127 094 | 147 135 | 70 721 | 577 516 |  | 206 669 | 303 272 | 306 670 | 534 906 | 479 612 | 1 831 129 |
| GINI 0.4 | 122 448 | 110 118 | 127 094 | 147 135 | 70 721 | 577 516 |  | 206 669 | 303 272 | 306 670 | 534 906 | 479 612 | 1 831 129 |
| Copayment out-of-pocket 20% | 85 206 | 77 340 | 89 044 | 103 852 | 50 392 | 405 835 |  | 122 289 | 179 451 | 181 462 | 316 512 | 283 794 | 1 083 508 |
| Copayment out-of-pocket 50% | 166 166 | 148 595 | 171 762 | 197 945 | 94 586 | 779 054 |  | 305 723 | 448 627 | 453 654 | 791 281 | 709 486 | 2 708 770 |
| Number of deaths due to ALRI -30% | 122 448 | 110 118 | 127 094 | 147 135 | 70 721 | 577 516 |  | 206 669 | 303 272 | 306 670 | 534 906 | 479 612 | 1 831 129 |
| Number of deaths due to ALRI +30% | 122 448 | 110 118 | 127 094 | 147 135 | 70 721 | 577 516 |  | 206 669 | 303 272 | 306 670 | 534 906 | 479 612 | 1 831 129 |
| Number of births and under 5 pop +20% | 144 627 | 130 533 | 150 512 | 174 750 | 84 307 | 684 730 |  | 248 003 | 363 926 | 368 004 | 641 887 | 575 535 | 2 197 354 |
| Number of births and under 5 pop -20% | 100 269 | 89 702 | 103 676 | 119 519 | 57 135 | 470 302 |  | 165 335 | 242 617 | 245 336 | 427 925 | 383 690 | 1 464 903 |
| Number of deaths due to SP -30% | 118 982 | 107 705 | 124 093 | 144 417 | 69 884 | 565 082 |  | 206 669 | 303 272 | 306 670 | 534 906 | 479 612 | 1 831 129 |
| Number of deaths due to SP +30% | 125 913 | 112 530 | 130 095 | 149 852 | 71 559 | 589 949 |  | 206 669 | 303 272 | 306 670 | 534 906 | 479 612 | 1 831 129 |

PCV=pneumococcal vaccine; DPT=Diphtheria-tetanus-pertussis-HepatitisB-Haemophilus influenzae type b; ALRI= Acute lower respiratory infection;

SP=Streptococcus pneumoniae
