# Supplementary material for: Health Gains and Financial Protection from Pneumococcal Vaccination and Pneumonia Treatment in Ethiopia: Results from an Extended Cost-Effectiveness Analysis
Source: PLoS One. 2015 Dec 9;10(12):e0142691. doi: 10.1371/journal.pone.0142691 (PMC4674114; doi:10.1371/journal.pone.0142691)
Supplement: S3 Table — (DOCX) [file pone.0142691.s003.docx]

**S3 Table:** Uncertainty analysis of the impact on deaths averted across income quintiles for each of the two policies in Ethiopia (pneumonia treatment and pneumococcal vaccines), key variables are modified as a one-way sensitivity analysis (Q1 is poorest and Q5 is richest; colours identify value variance, where black cells are the 10% highest values and grey cells are the 10% lowest values).

|  | **Pneumococcal vaccine** | | | | | |  | **Pneumonia treatment** | | | | | |
| --- | --- | --- | --- | --- | --- | --- | --- | --- | --- | --- | --- | --- | --- |
|  | **Q1** | **Q2** | **Q3** | **Q4** | **Q5** | **Total** |  | **Q1** | **Q2** | **Q3** | **Q4** | **Q5** | **Total** |
| Pneumonia treatment at 0% coverage |  |  |  |  |  |  |  | 0 | 0 | 0 | 0 | 0 | 0 |
| PCV 10% incremental coverage | **275** | **169** | **167** | **157** | **42** | **810** |  |  |  |  |  |  |  |
| **Pneumonia treatment 10% incremental coverage** | |  |  |  |  |  |  | 886 | 545 | 538 | 507 | 134 | 2 611 |
| **PCV coverage at DPT3 level** | 1 004 | 617 | 609 | 575 | 152 | 2 957 |  |  |  |  |  |  |  |
| PCV 80% incremental coverage | 2 200 | 1 353 | 1 335 | 1 259 | 334 | 6 481 |  |  |  |  |  |  |  |
| PCV 90% incremental coverage | 2 475 | 1 522 | 1 502 | 1 417 | 375 | 7 291 |  |  |  |  |  |  |  |
| Pneumonia treatment 80% incremental coverage | |  |  |  |  |  |  | 7 091 | 4 360 | 4 303 | 4 058 | 1 076 | 20 888 |
| Pneumonia treatment 90% incremental coverage | |  |  |  |  |  |  | 7 977 | 4 905 | 4 841 | 4 566 | 1 210 | 23 499 |
| PCV vial 0.2 US$ | 1 004 | 617 | 609 | 575 | 152 | 2 957 |  | 886 | 545 | 538 | 507 | 134 | 2 611 |
| PCV vial 1 US$ | 1 004 | 617 | 609 | 575 | 152 | 2 957 |  | 886 | 545 | 538 | 507 | 134 | 2 611 |
| Amoxicillin effect reduced to 0.6 | 1 004 | 617 | 609 | 575 | 152 | 2 957 |  | 760 | 467 | 461 | 435 | 115 | 2 238 |
| Amoxicillin effect increased to 0.8 | 1 004 | 617 | 609 | 575 | 152 | 2 957 |  | 1 013 | 623 | 615 | 580 | 154 | 2 984 |
| PCV effect reduced by 20% | 803 | 494 | 487 | 460 | 122 | 2 365 |  | 886 | 545 | 538 | 507 | 134 | 2 611 |
| PCV effect increased by 20% | 1 205 | 741 | 731 | 689 | 183 | 3 548 |  | 886 | 545 | 538 | 507 | 134 | 2 611 |
| 10% of those <5 years with pneumonia | 1 004 | 617 | 609 | 575 | 152 | 2 957 |  | 886 | 545 | 538 | 507 | 134 | 2 611 |
| 5% of those <5 years with pneumonia | 1 004 | 617 | 609 | 575 | 152 | 2 957 |  | 886 | 545 | 538 | 507 | 134 | 2 611 |
| GDP 300 US$ | 1 004 | 617 | 609 | 575 | 152 | 2 957 |  | 886 | 545 | 538 | 507 | 134 | 2 611 |
| GDP 400 US$ | 1 004 | 617 | 609 | 575 | 152 | 2 957 |  | 886 | 545 | 538 | 507 | 134 | 2 611 |
| GINI 0.2 | 1 004 | 617 | 609 | 575 | 152 | 2 957 |  | 886 | 545 | 538 | 507 | 134 | 2 611 |
| GINI 0.4 | 1 004 | 617 | 609 | 575 | 152 | 2 957 |  | 886 | 545 | 538 | 507 | 134 | 2 611 |
| Copayment out-of-pocket 20% | 1 004 | 617 | 609 | 575 | 152 | 2 957 |  | 886 | 545 | 538 | 507 | 134 | 2 611 |
| Copayment out-of-pocket 50% | 1 004 | 617 | 609 | 575 | 152 | 2 957 |  | 886 | 545 | 538 | 507 | 134 | 2 611 |
| Number of deaths due to ALRI -30% | 1 004 | 617 | 609 | 575 | 152 | 2 957 |  | 620 | 381 | 377 | 355 | 94 | 1 828 |
| Number of deaths due to ALRI +30% | 1 004 | 617 | 609 | 575 | 152 | 2 957 |  | 1 152 | 708 | 699 | 659 | 175 | 3 394 |
| Number of births and under 5 pop +20% | 1 004 | 617 | 609 | 575 | 152 | 2 957 |  | 886 | 545 | 538 | 507 | 134 | 2 611 |
| Number of births and under 5 pop -20% | 1 004 | 617 | 609 | 575 | 152 | 2 957 |  | 886 | 545 | 538 | 507 | 134 | 2 611 |
| Number of deaths due to SP -30% | 703 | 432 | 426 | 402 | 107 | 2 070 |  | 886 | 545 | 538 | 507 | 134 | 2 611 |
| Number of deaths due to SP +30% | 1 305 | 802 | 792 | 747 | 198 | 3 844 |  | 886 | 545 | 538 | 507 | 134 | 2 611 |

PCV=pneumococcal vaccine; DPT=Diphtheria-tetanus-pertussis-HepatitisB-Haemophilus influenzae type b; ALRI= Acute lower respiratory infection;

SP=Streptococcus pneumoniae
