# Supplementary material for: Health Gains and Financial Protection from Pneumococcal Vaccination and Pneumonia Treatment in Ethiopia: Results from an Extended Cost-Effectiveness Analysis
Source: PLoS One. 2015 Dec 9;10(12):e0142691. doi: 10.1371/journal.pone.0142691 (PMC4674114; doi:10.1371/journal.pone.0142691)
Supplement: S4 Table — (DOCX) [file pone.0142691.s004.docx]

**S4 Table:** Uncertainty analysis of the impact on financial risk protection (2011 US$) across income quintiles for each of the two policies in Ethiopia (pneumonia treatment and pneumococcal vaccines), key variables are modified as a one-way sensitivity analysis (Q1 is poorest and Q5 is richest; colours identify value variance, where black cells are the 10% highest values and grey cells are the 10% lowest values).

|  | **Pneumococcal vaccine** | | | | | |  | **Pneumonia treatment** | | | | | |
| --- | --- | --- | --- | --- | --- | --- | --- | --- | --- | --- | --- | --- | --- |
|  | **Q1** | **Q2** | **Q3** | **Q4** | **Q5** | **Total** |  | **Q1** | **Q2** | **Q3** | **Q4** | **Q5** | **Total** |
| Pneumonia treatment at 0% coverage |  |  |  |  |  |  |  | 102 141 | 35 801 | 29 168 | 22 950 | 6 686 | 196 746 |
| PCV 10% incremental coverage | **4 665** | **1 408** | **1 113** | **857** | **247** | **8 290** |  |  |  |  |  |  |  |
| **Pneumonia treatment 10% incremental coverage** | |  |  |  |  |  |  | 102 141 | 35 801 | 29 168 | 22 950 | 6 686 | 196 746 |
| **PCV coverage at DPT3 level** | 17 026 | 5 141 | 4 062 | 3 130 | 901 | 30 259 |  |  |  |  |  |  |  |
| PCV 80% incremental coverage | 37 317 | 11 268 | 8 903 | 6 860 | 1 974 | 66 321 |  |  |  |  |  |  |  |
| PCV 90% incremental coverage | 41 981 | 12 676 | 10 016 | 7 717 | 2 221 | 74 612 |  |  |  |  |  |  |  |
| Pneumonia treatment 80% incremental coverage | |  |  |  |  |  |  | 102 141 | 35 801 | 29 168 | 22 950 | 6 686 | 196 746 |
| Pneumonia treatment 90% incremental coverage | |  |  |  |  |  |  | 102 141 | 35 801 | 29 168 | 22 950 | 6 686 | 196 746 |
| PCV vial 0.2 US$ | 17 026 | 5 141 | 4 062 | 3 130 | 901 | 30 259 |  | 102 141 | 35 801 | 29 168 | 22 950 | 6 686 | 196 746 |
| PCV vial 1 US$ | 17 026 | 5 141 | 4 062 | 3 130 | 901 | 30 259 |  | 102 141 | 35 801 | 29 168 | 22 950 | 6 686 | 196 746 |
| Amoxicillin effect reduced to 0.6 | 17 026 | 5 141 | 4 062 | 3 130 | 901 | 30 259 |  | 102 141 | 35 801 | 29 168 | 22 950 | 6 686 | 196 746 |
| Amoxicillin effect increased to 0.8 | 17 026 | 5 141 | 4 062 | 3 130 | 901 | 30 259 |  | 102 141 | 35 801 | 29 168 | 22 950 | 6 686 | 196 746 |
| PCV effect reduced by 20% | 13 621 | 4 113 | 3 250 | 2 504 | 721 | 24 208 |  | 102 141 | 35 801 | 29 168 | 22 950 | 6 686 | 196 746 |
| PCV effect increased by 20% | 20 431 | 6 169 | 4 875 | 3 756 | 1 081 | 36 311 |  | 102 141 | 35 801 | 29 168 | 22 950 | 6 686 | 196 746 |
| 10% of those <5 years with pneumonia | 17 026 | 5 141 | 4 062 | 3 130 | 901 | 30 259 |  | 144 211 | 50 660 | 41 180 | 32 310 | 9 437 | 277 799 |
| 5% of those <5 years with pneumonia | 17 026 | 5 141 | 4 062 | 3 130 | 901 | 30 259 |  | 73 531 | 25 734 | 20 997 | 16 551 | 4 814 | 141 629 |
| GDP 300 US$ | 21 190 | 6 449 | 5 001 | 3 807 | 1 086 | 37 534 |  | 124 482 | 43 841 | 35 407 | 27 681 | 8 023 | 239 435 |
| GDP 400 US$ | 14 683 | 4 462 | 3 559 | 2 760 | 798 | 26 261 |  | 89 478 | 31 451 | 25 746 | 20 329 | 5 940 | 172 944 |
| GINI 0.2 | 8 554 | 4 095 | 3 774 | 3 265 | 1 070 | 20 759 |  | 56 009 | 29 078 | 27 226 | 23 905 | 7 915 | 144 133 |
| GINI 0.4 | 25 950 | 7 359 | 4 618 | 3 082 | 771 | 41 780 |  | 151 498 | 48 952 | 32 860 | 22 606 | 5 736 | 261 652 |
| Copayment out-of-pocket 20% | 6 966 | 2 262 | 1 828 | 1 461 | 438 | 12 955 |  | 31 976 | 11 821 | 9 798 | 7 811 | 2 301 | 63 706 |
| Copayment out-of-pocket 50% | 34 407 | 10 826 | 8 181 | 6 062 | 1 681 | 61 157 |  | 245 455 | 84 361 | 67 168 | 51 980 | 14 942 | 463 907 |
| Number of deaths due to ALRI -30% | 17 026 | 5 141 | 4 062 | 3 130 | 901 | 30 259 |  | 102 141 | 35 801 | 29 168 | 22 950 | 6 686 | 196 746 |
| Number of deaths due to ALRI +30% | 17 026 | 5 141 | 4 062 | 3 130 | 901 | 30 259 |  | 102 141 | 35 801 | 29 168 | 22 950 | 6 686 | 196 746 |
| Number of births and under 5 pop +20% | 19 603 | 5 973 | 4 723 | 3 661 | 1 063 | 35 024 |  | 122 569 | 42 962 | 35 002 | 27 540 | 8 024 | 236 095 |
| Number of births and under 5 pop -20% | 14 447 | 4 308 | 3 401 | 2 598 | 739 | 25 493 |  | 81 713 | 28 641 | 23 334 | 18 360 | 5 349 | 157 397 |
| Number of deaths due to SP -30% | 15 784 | 4 847 | 3 835 | 2 988 | 874 | 28 328 |  | 102 141 | 35 801 | 29 168 | 22 950 | 6 686 | 196 746 |
| Number of deaths due to SP +30% | 18 265 | 5 434 | 4 289 | 3 271 | 928 | 32 187 |  | 102 141 | 35 801 | 29 168 | 22 950 | 6 686 | 196 746 |

PCV=pneumococcal vaccine; DPT=Diphtheria-tetanus-pertussis-HepatitisB-Haemophilus influenzae type b; ALRI= Acute lower respiratory infection;

SP=Streptococcus pneumoniae
