# Supplementary material for: Health Gains and Financial Protection from Pneumococcal Vaccination and Pneumonia Treatment in Ethiopia: Results from an Extended Cost-Effectiveness Analysis
Source: PLoS One. 2015 Dec 9;10(12):e0142691. doi: 10.1371/journal.pone.0142691 (PMC4674114; doi:10.1371/journal.pone.0142691)
Supplement: S1 Text — (DOCX) [file pone.0142691.s005.docx]

**S1 Text. Supporting information**

1. **Methods for economic evaluation of public finance for pneumococcal vaccination and pneumonia treatment**

This section describes the methods we use for the economic evaluation of public finance for pneumococcal vaccination and pneumonia treatment in Ethiopia, drawing from methods described elsewhere [1,2]. Specifically, we estimate the level and distribution (across income groups) of:

(1) the burden of disease averted (deaths averted);

(2) the household private expenditures averted, and the total incremental costs of the program;

(3) the financial protection brought by the program measured by a monetary value of insurance provided.

In Ethiopia, we divide the population in five income groups *J*, and we define: *y*, the income of an individual, and *f(y)*, the income distribution.

*1.1. Pneumococcal diseases and pneumonia deaths averted*

We estimate the number of pneumococcal diseases and pneumonia deaths averted by the program for the under-five population in Ethiopia, using relative risks of under-five rotavirus mortality *RR_J_* derived from the Ethiopian Demographic and Health Survey [3,4] and explained in the Methods section of the main text. By income quintile *J*, the pneumococcal diseases and pneumonia deaths averted are:

$D_{post,J}=\frac{1}{5}{{RR}_{J}V_{eff}CovD}_{Total}$ (1)

where *D_Total_* is the total number of under-five pneumococcal diseases/pneumonia deaths in the country before the program, *V_eff_* is the intervention effectiveness, *Cov* is the intervention incremental coverage, and *RR_J_* is the relative risk of under-five pneumonia mortality for the income quintile *J*.

*1.2. Consequences for household private expenditures*

We estimate the household private expenditures averted (pneumococcal disease and pneumonia treatment costs averted by the program) in a given country.

For the income quintile *J*, the private expenditures averted by full public finance of vaccination (per capita) are given by:

${PE}_{vac,J}={V_{eff}Cov(p}_{inp,J}c_{inp,J}+p_{out,J}c_{out,J})$ (2)

where *p_inp,J_* and *p_out,J_* are the 5-year risks of inpatient and outpatient visits for pneumococcal diseases, respectively; *c_inp,J_* and *c_out,J_* are the out-of-pocket costs of inpatient and outpatient visit for pneumococcal diseases (transport costs to facility are also included in *c_inp,J_* and *c_out,J_*).

For the income quintile *J*, the private expenditures averted by full public finance of pneumonia treatment (per capita) are given by:

${PE}_{tr,J}={(p}_{inp,J}c_{inp,J}+p_{out,J}c_{out,J})$ (3)

where *p_inp,J_* and *p_out,J_* are the annual risks of inpatient and outpatient visits for pneumonia treatment, respectively; *c_inp,J_* and *c_out,J_* are the costs of inpatient and outpatient visit for pneumonia.

From the public sector perspective, total vaccination costs incurred (per capita) are:

${TC}_{PCV}=3Cov(c_{vaccine}+c_{program})$ (4)

where *c_vaccine_* and *c_program_* are the costs (per dose) of the vaccine and the program, respectively. The healthcare costs averted through vaccination (per capita, per quintile *J*) are:

${TC}_{HC,J}=Cov{V_{eff}(p}_{inp,J}c_{inp,gov,J}+p_{out,J}c_{out,gov,J})$ (5)

where $V_{eff}$ is the vaccine effectiveness against pneumococcal disease (either pneumonia, meningitis or non-pneumonia non-meningitis). *p_inp,J_* and *p_out,J_* are the 5-year risks of inpatient and outpatient visits for pneumococcal disease, respectively; *c_inp,gov,J_* and *c_out,gov,J_* are the government costs of inpatient and outpatient visit for pneumococcal disease, respectively (transport costs to facility are not included).

From the public sector perspective, total pneumonia treatment costs incurred (per capita, per quintile *J*) are:

${TC}_{tre,J}=Cov(c_{inp,J}+c_{out,J})+(p_{inp,J}c_{inp,J}+{p_{inp,J}c}_{out,J})(1-s)$ (6)

where *Cov* is the intervention incremental coverage, *p_inp,J_* and *p_out,J_* are the annual risks of inpatient and outpatient visits for pneumonia treatment, respectively; *c_inp,J_* and *c_out,J_* are the costs of inpatient and outpatient visit for pneumonia; and $s$ is the share of pneumonia treatment cost covered by the government before pneumonia treatment scale-up (assumed to be$s=0.66$ here).

*1.3. Monetary value of insurance*

We apply a utility-based model where risk-averse individuals value protection from the risk of rare events, and we estimate the expected value of the gamble associated with the eventuality of the disease with probability *p* and treatment cost *c*, before the program is introduced [1,2,5].

The expected value to an individual of income *y* in the quintile *J* of the gamble with the disease prior to the program is:

$E_{J}(y)=p_{inp,J}\left( y-c_{inp,J} \right)+p_{out,J}\left( y-c_{out,J} \right)$

$+\left( 1-p_{inp,J}-p_{out,J} \right)y$ (7)

The certainty equivalent for the same individual, noted *Y_J_**, is:

$Y_{J}^{*}= U^{-1}[p_{inp,J}U\left( y-c_{inp,J} \right)+p_{out,J}U\left( y-c_{out,J} \right)$

$+\left( 1-p_{inp,J}-p_{out,J} \right)U(y)$] (8)

where *U* is a constant relative risk aversion (CRRA) utility function:

$U\left( y \right)=\frac{y^{1-r}}{1-r}$ (9)

with *r* the coefficient of relative risk aversion (here *r* = 3 [1,2,5]). The monetary value of the insurance for the individual of income *y* in the quintile *J* would then be:

$E_{J}(y)-Y_{J}^{*}$ (10)

For pneumococcal vaccination, as only a fraction of the population (*Cov*) receives the intervention of efficacy *V_eff_*, the total monetary value of insurance for the quintile *J* is:

${Ins}_{J}=V_{eff}Cov\int_{J} (E_{J}(y)-Y_{J}^{*})f\left( y \right)dy.$ (11)

where $f(y)$ is the income distribution in the population proxied by a Gamma density based on country gross domestic product per capita and Gini index [6,7].

*1.4. Methods for estimating the introduction costs of strengthened routine immunization*

This section describes the methods we use for estimating the introduction costs of strengthening the routine immunization program in Ethiopia. Introduction cost for each vaccine is calculated as:

$I_{cost}=n_{birts}{Cov}_{Total}n\_injections(Cost_{sys}+Cost_{vial})$ (12)

where *n_births_* is the total annual number of births in Ethiopia, Cov*_Total_* is the target vaccine coverage, n*_injections_* is the number of doses per course, *Cost_sys_* is average supply cost per dose (including cold storage, transport, training and public communication), and Cost*_vial_* is the price per vaccine vial. We assumed that incremental cost by going from current coverage to a 90% target level entailed a twofold *Cost_sys in_* to adjust for the fact that more investments are needed into the system to cover hard to reach groups.

*1.5. Uncertainty analysis*

We apply a one-way deterministic sensitivity analysis to explore variations in results according to parameter assumptions in the model. Around a 20-30% increase or reduction of all variables are introduced, and detailed results of this sensitivity analysis are presented in S2-S4 Tables.

**References**

[1] Verguet S, Laxminarayan R, Jamison DT. Universal public finance of turberculosis treatment in India: an extended cost-effectiveness analysis. Health Economics 2015; 24(3):318-322.

[2] Verguet S, Murphy S, Anderson B, Johansson KA, Glass R, et al. (2013) Public finance of rotavirus vaccination in India and Ethiopia: An extended cost-effectiveness analysis. Vaccine 31: 4902-4910.

[3] Rheingans RD, Atherly D, Anderson J. Distributional impact of rotavirus vaccination in 25 GAVI countries: estimating disparities in benefits and cost-effectiveness. Vaccine 2012; 30S:A15-A23.

[4] Central Statistical Agency [Ethiopia], ICF International (2011) Ethiopia Demographic and Health Survey. Addis Ababa, Ethiopia and Calverton, Maryland, USA: Central Statistical Agency and ICF International.

[5] McClellan M, Skinner J. The incidence of Medicare. Journal of Public Economics 2006;

90: 257-276.

[6] Salem ABZ, Mount TD. A Convenient Descriptive Model of Income Distribution: The Gamma Density, Econometrica 1974; 42(6):1115-1127.

[7] World Bank, World Development Indicators (2014): The World Bank: Washington, DC.
